# Current Concepts in Management of Acromioclavicular Joint Injury

**DOI:** 10.3390/jcm13051413

**Published:** 2024-02-29

**Authors:** Carter M. Lindborg, Richard D. Smith, Alec M. Reihl, Blake M. Bacevich, Mark Cote, Evan O’Donnell, Augustus D. Mazzocca, Ian Hutchinson

**Affiliations:** 1 Department of Orthopaedic Surgery, Massachusetts General Hospital, Harvard Medical School, Boston, MA 02115, USA; cmlindborg@mgh.harvard.edu (C.M.L.); rsmith21@mgb.org (R.D.S.); areihl@bwh.harvard.edu (A.M.R.); blakebacevich@hms.harvard.edu (B.M.B.); eodonnell4@mgb.org (E.O.); amazzocca@mgh.harvard.edu (A.D.M.); 2 Department of Orthopaedic Surgery, University of Connecticut Health Center, Farmington, CT 06030, USA; mcote2@mgh.harvard.edu

**Keywords:** acromioclavicular joint, anatomy, acromioclavicular injury, anatomic coracoclavicular reconstruction, narrative review

## Abstract

**Background**: The management of acromioclavicular joint injuries requires a thorough understanding of the anatomy and biomechanics of the joint, as well as knowledge of the pertinent physical exam findings and classification to determine an appropriate treatment approach, whether operative or nonoperative. In this article, we present a narrative review of the current state of understanding surrounding these issues. Although there are a large number of options for operative intervention, we additionally present our experience with anatomic coracoclavicular ligament reconstruction (ACCR) with imbrication of the deltoid fascia. **Methods**: A retrospective review of prospectively collected data on a total of 45 patients who had undergone ACCR between 2003 and 2016 were collected. **Results**: We found that improvements were seen in American Shoulder and Elbow Surgeons Score (ASES) (53 ± 19 to 81 ± 23), Simple Shoulder Test (SST) (6 ± 3 to 12 ± 13), Constant–Murley (CM) (60 ± 18 to 92 ± 8), and Rowe (67 ± 14 to 89 ± 11) and the mean post-operative SANE score was 86 ± 17. **Conclusions**: ACCR has the advantage of addressing both horizontal and vertical stability with good outcomes.

## 1. Anatomy and Biomechanics: Implications for Management of AC Injuries

The management of acromioclavicular (AC) joint injuries in orthopedic practice is heavily influenced by a comprehensive understanding of the joint’s unique anatomy and biomechanics. The intricate balance between the anatomy of the AC joint and the functional demands placed upon it informs the entire spectrum of injury management, from initial assessment to definitive treatment and rehabilitation.

Anatomically, the AC joint is a diarthrodial, synovial joint formed by the articulation between the distal clavicle and the medial aspect of the acromion of the scapula. This joint allows for complex osteokinematic movements, including flexion, extension, and rotation of the arm, which are critical for tasks ranging from simple daily activities to high-performance athletic maneuvers. Underlying these apparent movements are the arthrokinematic processes of the joint, characterized by subtle gliding and rotating actions between the distal clavicle and the acromion of the scapula. These micro-movements are crucial for the smooth and coordinated motion of the shoulder complex. Facilitating these osteokinematic and arthrokinematic movements, the joint is supported by both static and dynamic stabilizers, with static stabilization provided by the AC capsuloligamentous structures and coracoclavicular (CC) ligaments (conoid and trapezoid) [1,2], contrary to the classical conceptualization of the AC ligaments into two distinct bundles: the superoposterior (SP) bundle and the anteroinferior (AI) bundle [3,4]. While both bundles contribute to static stabilization, the SP bundle runs at a more oblique angle with respect to the distal third of the clavicle [3,5]. The SP bundle has also been demonstrated to have a much more diverse insertional footprint on both the clavicle and acromion than the AI bundle [5] While both bundles primarily stabilize against horizontal forces and resist anteroposterior and superior displacement of the clavicle, recent data from Kurata et al. suggest that the SP bundle provides significantly more support than the AI bundle [6]. In contrast, the CC ligaments primarily provide vertical static stability by connecting the base of the coracoid and the distal third of the clavicle. The conoid ligament, cone-shaped and located posteromedially, resists axial compression forces, while the trapezoid ligament, quadrilateral-shaped and situated more anterolaterally, is crucial in preventing both superior and posterior displacement of the clavicle [7,8,9].

In addition to static stabilizers, the AC joint is further supported by a number of dynamic stabilizers made up of the deltoid, trapezius, and deltotrapezial fascia [2,10]. The contraction of the upper trapezius muscle generates a force that is responsible for the retraction and elevation of the distal clavicle. Similarly, the force produced by middle trapezius muscle contraction leads to the retraction of the scapula. These actions may alleviate the stress placed on the static AC capsuloligamentous structures and CC ligaments during thoracohumeral motion [11]. While this may have significant implications for protecting reconstructed ligaments, the synergistic effect of the static and dynamic AC joint stabilizers is still a relatively new concept and is currently poorly understood within the orthopedic community.

Complementing the role of these stabilizers is the articular cartilage lining the surfaces of the distal clavicle and the acromion. This fibrocartilage lining facilitates smooth movement between these bones, reducing friction and absorbing shock during shoulder movements. The thickness and quality of this cartilage vary among individuals, influencing the joint’s susceptibility to wear and degeneration [12]. Additionally, the presence of a meniscal variant, or a fibrocartilaginous disc within the joint, is observed in some individuals [13]. However, it is noteworthy that this disc is not deemed necessary for joint function and often undergoes degeneration early in the second decade of life, rendering it nonfunctional in most adults [14]. Yet, despite its tendency to degenerate, the size and shape of this structure in younger individuals can significantly affect the joint’s biomechanics, leading to altered load distribution across the articular surfaces. This alteration can cause uneven wear and increased stress in specific cartilage areas, potentially predisposing the joint to osteoarthritis or exacerbating conditions such as impingement syndromes [15].

The anatomical and biomechanical variability of the AC joint plays a pivotal role in clinical decision making due to its direct influence on joint functionality and vulnerability to injury. For example, clavicular morphology, including the degree of curvature and length, has demonstrated notable variations [16]. These anatomical differences can potentially influence the biomechanics of the shoulder, including the lever-arm forces exerted on the AC joint. Additionally, variations in the size and orientation of the acromion, such as a low lateral acromial angle or a large lateral extension, can affect the joint space and the likelihood of impingement [17]. Furthermore, individual differences in the shape and depth of the articular facets of the clavicle and acromion may influence how these bones articulate, possibly affecting the joint’s overall congruency and range of motion [18]. While the direct relationship between these anatomical characteristics and the risk of joint stress or injury requires further investigation, understanding these variations is important in assessing individual susceptibility to shoulder injuries.

Detailed anatomical knowledge of the AC joint Is also important in planning rehabilitation strategies following injury. The dynamic stabilizers, particularly the deltoid and trapezius muscles, play a key role in compensating for the loss of ligamentous support after such injuries [11]. While specific strategies may vary based on individual patient needs and the nature of the injury, a focus on enhancing the strength and function of these muscles could be beneficial in both conservative and post-operative management of AC joint injuries. For instance, patients with well-developed deltoid and trapezius muscles might be candidates for more aggressive rehabilitation strategies, including early mobilization, scapular stabilization, and enhancing dynamic joint stability. Conversely, patients with less developed periscapular musculature may benefit from a gradual approach, focusing on progressive strengthening and stabilization exercises to mitigate the risk of re-injury.

From the variable strength and orientation of the stabilizing ligaments to the joint congruency and the surrounding musculature’s conditioning, each aspect of the AC joint’s anatomy and biomechanics plays a pivotal role in enabling surgeons to tailor their management plan to each individual patient. As research continues to evolve, this anatomically informed approach will continue to refine and enhance the care provided to patients with AC joint injuries.

## 2. Pearls of Physical Exam

AC injuries most often are the result of a direct fall onto the outer aspect of the shoulder, forcing the acromion inferiorly and rupturing the acromioclavicular ligaments with continued force transmission resulting in failure of the coracoclavicular ligaments [19]. AC injuries may be difficult to distinguish as pain over the superior–anterior shoulder can be a result of irritation to various structures. To further complicate the clinical picture, pathology of the AC joint may result in pain over the anterolateral deltoid region, supraspinatus, and the trapezius and also has been described as radiating into the anterolateral neck [20]. A complete and thorough physical exam is crucial in the identification of AC pathology, keeping in mind that AC injuries may affect the function of the glenohumeral joint and the scapulothoracic joint. To begin the exam, a visual inspection and then palpation are performed, identifying all of the exterior landmarks of the shoulder, noting areas of tenderness and deformity. A complete neurovascular exam of the extremity is also vital as neurologic injury can be associated with AC injuries [1].

There are a number of exam maneuvers that if positive demonstrate pain specific to the AC joint. The cross-arm adduction test is a provocative movement in which the patient elevates the arm to 90°, and with the elbow bent to 90°, the arm is adducted across the chest. This causes compression and elicits pain in the pathologic AC joint [21]. The cross-body adduction test is a fairly reliable maneuver, with Chronopoukos et al. reporting a sensitivity of 77% and a specificity of 79% in the identification of chronic AC joint pathology [22]. To perform the O’Brien active compression test, patients must flex their arm to 90° with the elbow at full extension and adduct the arm to approximately 15° with the thumb pointed toward the ground. Downward pressure is then applied to the arm from the examiner who is standing behind the patient. The patient then is asked to fully supinate the hand and the same pressure is applied. The test is considered positive if there is pain with the thumb pointed down and the pain is relieved when the patient is supinated [23]. The Paxinos sign is an additional tool when examining the AC joint. The examiner applies squeezing pressure across the AC joint with their thumb on the posterior lateral aspect of the acromion and their index and middle finger superior to the clavicle. The Paxinos sign is considered positive if this maneuver elicits pain. Using these tests in combination is the most accurate way to diagnose AC pathology, with Krill et al. reporting a specificity of 95.8% when the Paxinos sign and O’Brien compression test are performed together [24]. It is important to understand that these tests assist with helping to identify the pathology of the AC joint but may be positive in disease processes other than AC separation, including chronic or degenerative disease processes.

Assessing clavicular stability after AC injury is key to understanding the severity of the injury. The physical exam is the first step in AC injury classification but must be considered alongside radiographic findings. A displaced acromion indicates injury to the acromioclavicular and the coracoclavicular ligaments and is indicative of AC instability and a severe injury (Rockwood type III–VI) [25]. If horizontal, vertical, or rotational instability is present it may result in scapular dysmotility and pain [1]. Vertical instability is suggested by a visible deformity of the acromion which may be inferiorly dislocated. Tauber et al. discuss assessing for horizontal instability by holding the acromion static with one hand and attempting to translate the distal clavicle posteriorly [26]. Increased posterior translation compared to the uninjured side indicates that the AC joint is horizontally unstable. In Rockwood type IV injuries, deformity may be noted over the posterior shoulder as the clavicle can be palpated as it bulges through the trapezius, whereas in Rockwood type V injuries, the clavicle may tent the skin superiorly due to injury of the deltoid and trapezius fascia with an increase in the CC distance of 2–3 times [1,19]. The shrug test is a special test indicated in vertically unstable AC joints. To perform this test the examiner simply asks the patient to shrug their shoulders. If the vertically unstable AC joint reduces with this maneuver, it is indicative of a Rockwood type III injury. If the patient remains dislocated during the shrug, this is indicative of a type V injury [27].

As the scapulothoracic joint is closely associated with the AC joint, injuries to the AC joint may cause scapular dyskinesis [28]. Scapular dyskinesis describes an abnormal motion, position, and rhythm of the scapula, which can include both scapular winging and popping. Scapulohumeral rhythm is key to the proper function of the ball and socket joint and if disrupted may cause dysfunction of the arm and shoulder, including decreased rotator cuff strength and symptoms of impingement [29]. The scapulothoracic joint should be examined approximately 10 days post-injury when the pain decreases to allow for proper examination. The examiner stands behind the patient with the resting position of the scapula noted. The scapular motion and position of the medial border of the scapula should also be assessed during flexion and extension of the arm. If the AC joint is dislocated, it should be reduced and the scapula reexamined [30]. Special tests that may assist in the evaluation for scapular dyskinesis include the scapular assistance test and the scapular retraction test. The scapular assistance test is completed by applying pressure over the patient’s inferior medial scapula with the examiner’s other hand placed with the palm on the superior scapula and fingers applying pressure to the clavicle. In this position, the patient is asked to elevate the arm in the scapular plane while the examiner applies inferior pressure to the superior clavicle and then upward and laterally with the inferior hand to simulate the normal motion of the scapula. The test is positive if the patient has a reduction in pain with the maneuver compared to the elevation without any assistance [31]. The scapular retraction test is a test of supraspinatus strength after scapular stabilization. The examiner performs an empty can test by having the patient raise the injured arm in the scapular plane and point the thumb to the ground while pushing upward against the downward pressure on the arm applied by the examiner. This test is then repeated with the examiner stabilizing the scapula with their forearm on the medial border of the scapula [32]. The test is considered positive if the patient demonstrates increased supraspinatus strength after scapular stabilization. A thorough scapular exam plays a crucial role in the evaluation of AC joint injuries as the presence of scapular dyskinesis may indicate the need for operative management, specifically in patients with Rockwood type III AC injuries [30,33].

## 3. Management of Acromioclavicular Joint Injuries

### 3.1. Classification Systems

Management of acromioclavicular joint (ACJ) injuries depends upon the injury type. In the 1960s, Tossy et al. [34] and Allman [35] developed the first ACJ injury classifications. In 1984, the Rockwood classification was created, which contains six types based on which ligaments are injured, in addition to the degree and direction of clavicle displacement [36].

### 3.2. Type I and Type II Injuries: Nonoperative Management

A Rockwood type I injury is a sprain of the AC ligaments [36], and typically requires 1–2 weeks of sling for comfort. A retrospective study by Park et al. found patients with type I injuries were immobilized for an average of 19.5 days, with some patients still experiencing symptoms 6 weeks after injury [37].

A Rockwood type II injury involves rupture of the AC ligaments [36]. Management involves 3–4 weeks of sling for comfort. Park et al. found patients with type II injuries were immobilized for an average of 27 days, with some patients also experiencing symptoms up to 6 weeks after injury [37].

While most patients respond well to nonoperative management of types I and II injuries [38], some experience persistent symptoms. Mikek et al. studied 23 patients with type I and II injuries 10.2 years after injury and found that 52% reported at least occasional symptoms [39]. In a study of 33 patients with type II injuries, at an average 6.2-year follow-up, 48% had persistent symptoms and 27% went on to receive surgery [40]. For patients with type II injuries who have persistent symptoms after a trial of nonoperative management, distal clavicle resection may offer symptomatic relief [41].

### 3.3. Type III Injuries: Nonoperative Management

Rockwood type III injuries involve rupture of the AC and CC ligaments, with superior displacement up to 100% of the clavicle width [36]. While there is a lack of consensus regarding definitive management of these injuries, most patients should undergo an initial trial of nonoperative management with sling immobilization for 4–5 weeks [42].

A randomized controlled trial (RCT) comparing operative and nonoperative management of types III and V injuries found no difference in clinical outcomes at 18–20 years follow-up [43]. An RCT compared hookplate operative management with nonoperative management of types III and V injuries and found no difference in clinical outcomes at 24 months [44]. Another RCT found equivalent clinical outcomes at 1 year for suspensory operative fixation and nonoperative management of type III and IV injuries [45]. However, the nonoperative group had earlier return to work, higher 6-week DASH scores, and lower cost of care [45].

Two meta-analyses comparing nonoperative to operative management of type III injuries found no difference in clinical outcomes [46,47]. While the operative group showed improved cosmesis, they also experienced a longer duration of sick leave [46,47]. A systematic review examining type III injuries found equivalent clinical outcomes of patients managed operatively and nonoperatively, with a quicker return to work and sport for the nonoperative group [48]. Surveys have found that most surgeons manage type III injuries nonoperatively in the Netherlands [49], United Kingdom [50], and the United States [51,52].

While nonoperative management of type III injuries often yields satisfactory outcomes, this is not universally true. A prospective evaluation of 25 patients with type III injuries managed nonoperatively found no limitation in shoulder motion and rotational strength [53]. However, there was a 17% decrease in bench-press strength and 16% of patients deemed the outcome suboptimal [53]. In a study involving 34 patients with chronic type III injuries, 70.6% had scapular dyskinesis, of which 58% were diagnosed with SICK (Scapular malposition, Inferior medial border prominence, Coracoid pain and malposition, and dyskinesis of scapular movement) scapula syndrome [28]. Furthermore, a network meta-analysis of RCTs for the treatment of types III-V suggested operative management led to superior outcomes compared to nonoperative management [54].

### 3.4. Type III Injuries: Operative Management

Some studies report favorable outcomes with surgical management of type III injuries. In a retrospective study of 24 patients treated with a hook plate compared to 17 patients treated nonoperatively for type III injuries, operative management yielded superior outcomes [55]. A review of 46 patients with type III injuries treated operatively reported that at 21-year follow-up, 92% had satisfactory outcomes and would choose surgical treatment again [56].

### 3.5. Type IV, V, and VI Injuries: Nonoperative Management

Rockwood type IV injuries involves posterior displacement of the clavicle, type V injuries involve more than 100% superior displacement of the clavicle, and type VI injuries involve inferior displacement of the clavicle, which becomes trapped behind the conjoined tendon [36]. While some surgeons advocate for operative management of injury types IV, V, and VI, some studies have reported favorable outcomes with nonoperative management. An RCT comparing operative and nonoperative management of types III, IV, and V injuries found no difference in clinical outcomes [57]. However, the operative group experienced more complications (seven major and seven minor) compared to the nonoperative group (two major and one minor) [57]. A systematic review and meta-analysis of type III-V injuries showed no clinical differences in functional outcomes between operative and nonoperative management [58]. A retrospective review of 22 patients with type V injuries managed nonoperatively found that at an average of 34 months, most patients had returned to work but reported lower patient outcome scores [59].

### 3.6. Type IV, V, and VI Injuries: Operative Management

Most surgeons recommend operative management for injury types IV, V, and VI [60,61]. A systematic review found an almost perfect rate of return to sport (94–100%) in patients with types III, IV, and V injuries managed operatively [62]. An epidemiological study on members of the US military academy found that 71% of patients with types III-VI injuries were managed operatively and returned to their previous level of activity [63]. A retrospective study of 17 patients with type III, IV, and V injuries managed operatively had significant improvement in ASES score and pain post-operatively [64]. Another retrospective review of patients with type IV injuries found superior clinical outcomes in operative management compared to nonoperative management [65].

However, superior outcomes with surgical management of types IV, V, and VI are not unanimous. A prospective study of operatively treated type V injuries with a mean follow-up of 5.1 years found fair to poor outcomes in 28.6% of patients with 40% loss of surgical reduction [66]. Chen et al. reported a relatively high complication rate of 35% of patients treated operatively for types III, IV, and V injuries [67].

## 4. Surgical Techniques

There is a wide array of described surgical interventions for ACJ reconstruction, without a clearly defined gold standard. Indicative of this lack of a gold standard, over 150 surgical techniques have been described for ACJ reconstruction [48]. These 150 plus techniques can be divided into open or arthroscopic-assisted, which may be either anatomic or nonanatomic. Anatomic reconstruction techniques focus on the goal of reproduction of both the conoid and trapezoid ligaments, while nonanatomic techniques consist of either a single coracoclavicular ligament or open reduction and internal fixation of the joint with hardware [48]. An important consideration when choosing a surgical technique is the time from injury. Early intervention within 3 weeks and before the CC ligaments have diminished affords the opportunity for interventions such as the use of Kirschner wires or a hook plate, which take advantage of the improved capacity for healing of these ligaments [48]. This limited time for intervention can present a challenge as it can take 2 weeks to accurately assess scapular dyskinesis and the patient’s ability to cope with their injury. After 3 weeks, augmentation with a biologic or synthetic graft is needed to reconstruct the deficient ligaments and avoid superior migration.

### 4.1. Open Non-Anatomic Technique

Open techniques for ACJ reconstruction date back as far as 1861 when Samuel Cooper used a wire loop to repair the AC joint. In 1886, Baum became the first to combine repair of both the AC and CC ligaments and in 1917, Delbet described using a suture loop around the coracoid that was fixed in clavicular tunnels [48]. Cadenet, also in 1917, described coracoacromial ligament and tendon transfer to manage dislocations and fractures of the outer end of the clavicle [68]. Weaver and Dunn modified Cadenet’s technique in the early 1950s and used it for both acute and chronic type III injuries. In 1972, Weaver and Dunn published their technique involving distal clavicle excision combined with transfer of the CA ligament from the acromion to the remains of the clavicle [69]. In their initial report, only 75% of patients had good to excellent outcomes. The Weaver and Dunn procedure has been associated with a high rate of deformity recurrence due to relative weakness of the construct and continued AP instability.

Bosworth initially described coracoclavicular lag-screw fixation in 1941. As initially described, the AC joint was opened and reduced and subsequently a screw was inserted through the distal clavicle engaging the coracoid process [70]. Initial studies reported good long-term outcomes. However, later studies showed a high failure rate and the use of this technique declined.

Hook plates were first described in 1976 by Balser [71]. The plate is fixed to the superior aspect of the clavicle and a transarticular hook engages the inferior surface of the acromion to maintain the reduction. Modifications have been described including the addition of direct CC ligament repair, suturing of the capsuloligamentous complex, additional screw fixation, as well as augmentation and tendon transfers [1]. Many studies report good outcomes with use of the hook plate including frequent reports of early return to work and sport. Complications are not uncommon and the plate should be removed 3-months post-operation. Some reported complications with hook plates include loss of reduction following plate removal, subacromial impingement, and acromial erosion due to the hook [1].

While Kirschner wires have been described as another form of primary acromioclavicular joint fixation, this method of fixation has a high rate of hardware complications including migration and breakage of the pins [43,72].

### 4.2. Open Anatomic Technique

One of the first descriptions of an anatomic CC ligament reconstruction was published in 1928 by Bunnell, who incorporated a fascial graft weave to stabilize both the clavicle and scapula [73]. Over the last few decades, the use of autograft or allograft for anatomic ACJ reconstruction has grown. This method typically involves passing the graft through tunnels in the clavicle and around the coracoid and then securing the graft to the clavicle. Some of the complications associated with this technique are graft rupture, coracoid or clavicular fractures, and adhesive capsulitis [74].

### 4.3. Arthroscopic-Assisted Techniques

Arthroscopic techniques have purported advantages of less soft tissue dissection and the ability to concomitantly treat intra-articular pathology and preserve normal joint motion. There are numerous described methods including the dog bone button [75] and the TightRope system, with single or double systems [76]. Suture augmentation and synthetic devices can be extraosseous, intraosseous, or transosseous [1]. These techniques rely on the suture material acting as an internal augmentation to the repair while ligamentous healing occurs. This may be insufficient in the chronic and revision setting and biologic augmentation may be necessitated in these situations.

## 5. Case Series

The authors’ preferred technique is initial nonoperative management followed by open anatomic coracoclavicular ligament reconstruction (ACCR) with imbrication of the deltoid fascia. ACCR was performed as previously described in the literature. Briefly this consists of reconstruction of the coracoclavicular ligaments with a semitendinosus allograft which is passed beneath the coracoid and through bone tunnels in the clavicle and subsequently secured with interference screw fixation while retaining the acromioclavicular joint [77]. The lateral limb is left 2 cm longer and sutured to the posterior acromial tissue to recreate the AC joint capsule [78]. The deltoid fascia is then brought over the top and secured with an anchor anteriorly [79]. A retrospective review of prospectively collected data on patients undergoing ACCRs performed by the senior author was conducted. Of 70 retrospectively identified cases performed between 2003 and 2016, 9 were excluded as revisions, 16 were excluded for missing follow up data and a total of 45 patients were analyzed (follow up rate for primary surgeries 74%). These 45 patients had an average age of 43 (±13 Standard deviation (SD)) years, and 7 (16%) were female. The average duration of follow up was 35 months (±29 SD).

### Results

Patients were evaluated pre- and post-operatively (Table 1) and the American Shoulder and Elbow Surgeons Score (ASES), the Simple Shoulder Test (SST), the Constant Murley (CM) Score, and the Rowe shoulder instability score were collected. The Self Assessment Numeric Evaluation (SANE) score was also collected post-operatively. Studies of acromioclavicular joint reconstruction have previously used mean clinically important difference (MCID), substantial clinical benefit (SCB), and patient acceptable symptomatic states (PASS) as defined for rotator cuff tears of 11, 17.5, and ASES of 86.7 for PASS [79,80]. A 2-tailed t-test was performed to assess for significance of improvement. Improvements were seen in ASES (53 ± 19 to 81 ± 23, *p* < 0.0001) (mean ± SD, *p*), SST (6 ± 3 to 12 ± 13, 0.0033), CM (60 ± 18 to 92 ± 8, *p* < 0.0001), and Rowe (67 ± 14 to 89 ± 11, *p* < 0.0001). The mean post-operative SANE score was 86 ± 17. Table 2 shows a breakdown of the post-operative SANE scores. In total, 70% of patients achieved a SANE score greater than 80, while 15% scored 70–80 and 15% scored < 70.

This review has several limitations. There was a lack of long-term radiological follow up to assess for re-subluxation, as well as a lack of data availability on long-term rates of reoperation or complications.

## 6. Conclusions

Effective management of ACJ injuries requires a thorough understanding of the anatomy and biomechanics of the AC joint, including the static and dynamic stabilizers and their implications for both operative and nonoperative management, as well as thoughtful evaluation of patient-specific factors including recreational and occupational physical activity, age, cosmetic concerns, symptom duration, and severity. The greatest uncertainty surrounds the management of type III injuries. A large multi-center RCT comparing operative and nonoperative management is required, incorporating the use of new surgical techniques to help guide the future. While there is no gold standard consensus for management or surgical techniques, ACCR with fascial imbrication provides an anatomic reduction and addresses both vertical and horizontal instability with good results.

## Figures and Tables

**Table 1:** Pre- and post-operative outcomes.

	*Pre* (mean ± SD)	*Post* (mean ± SD)	*Difference* (mean ± SD)	*p*
Rowe	67 (±14)	89 (±11)	21 (±11)	<0.0001
ASES	53 (±19)	81 (±23)	31 (±21)	<0.0001
SST	6 (±3)	12 (±13)	5 (±13)	0.0033
CM	60 (±18)	92 (±8)	26 (±12)	<0.0001
SANE		86 (±17)		

**Table 2:** Distribution of SANE scores.

SANE Score	% of Patients
<70	15%
70–80	15%
81–90	20%
91–100	50%

## Data Availability

The data presented in this study are available on request from the corresponding author. The data are not publicly available due to restrictions regarding privacy.
